# The Three P's: Parotid, PD-L1, and Pembrolizumab

**DOI:** 10.1155/2019/2305315

**Published:** 2019-06-11

**Authors:** Amanda Wiggins, Zhaohui Arter, Tamie Kerns

**Affiliations:** Tripler Army Medical Center, 1 Jarrett White Road, DHCK-DM, Honolulu, HI 96859, USA

## Abstract

We present a case of recurrent, platinum-refractory undifferentiated carcinoma of the parotid which was treated with checkpoint inhibitor, Pembrolizumab, and achieved a complete response to therapy. We review the literature of checkpoint inhibitor use in undifferentiated carcinoma of the parotid.

## 1. Introduction

Immunologic therapy is an emerging treatment modality in oncology, which may provide prolonged survival in numerous malignancies [1]. PD-L1 expression has been found to have both prognostic and treatment utility in the management of metastatic malignancies [2]. We present a patient with one hundred percent PD-L1 expression in an undifferentiated, EBV positive, metastatic carcinoma of parotid origin with near complete remission after treatment with Pembrolizumab.

## 2. Case Presentation

A previously healthy 52-year-old Samoan woman initially presented to her primary care provider with complaints of otalgia and swelling in the left side of her face for three weeks. She reported no facial weakness, trismus, dysphagia, odynophagia, dental pain, fevers, chills, weight loss, and fatigue. On initial physical exam, a 6 cm nontender, subcutaneous, cystic mass was palpated in the left parotid. The oral cavity showed no deformities or evidence of abnormalities. There was no lymphadenopathy of the anterior or posterior cervical chain, supraclavicular, or axillary lymph nodes. At that time, she was prescribed with antibiotics for presumed sialadenitis with no effect on her symptoms. On the next follow-up visit, she was referred to otolaryngology for further evaluation.

A neck and chest computer topography (CT) scan demonstrated two necrotic left parotid masses measuring 2.5 × 2.8 cm and 2.7 × 2.8 cm, respectively, multiple ipsilateral lymph nodes measuring up to 1.9 cm in diameter, and an asymmetrically enhancing left nasopharynx. A fine needle aspiration (FNA) of an involved local lymph node revealed a nonkeratinizing, undifferentiated carcinoma composed of pleomorphic cells positive for Epstein-Barr virus (EBV). The differential diagnosis based on FNA findings includes primary parotid carcinoma, lymphoepithelial carcinoma, or metastatic nasopharyngeal carcinoma. Blind biopsies of the nasopharynx were negative. PET/CT revealed hypermetabolic activity in the left parotid gland and several local nodes, highly suggestive of a primary parotid neoplasm. Excisional biopsy revealed a nonkeratinizing, undifferentiated carcinoma composed of pleomorphic cells, positive for Epstein-Barr virus (EBV). The results of subsequent excisional biopsy of the parotid gland masses were consistent with previous FNA findings.

**Figure 1:**
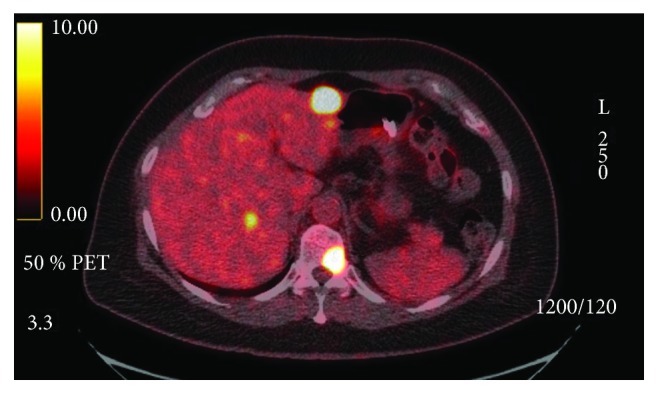
PET/CT image prior to immunotherapy.

**Figure 2:**
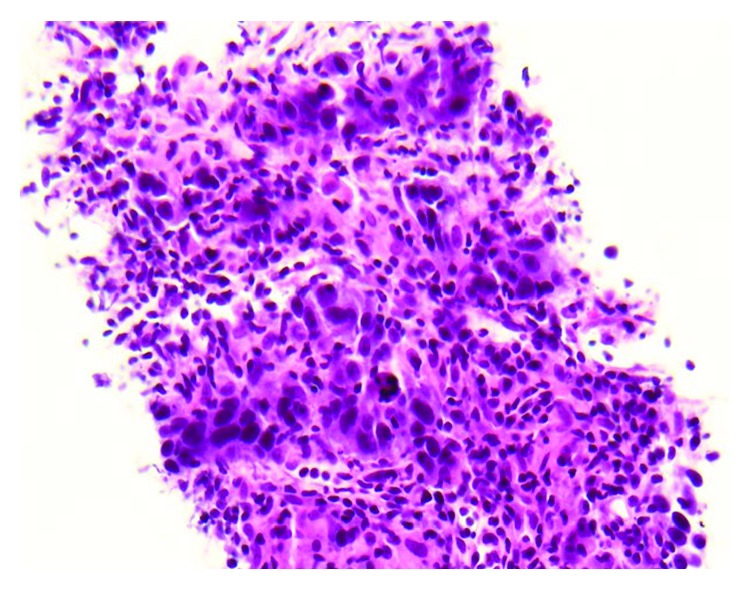
Biopsy of metastatic lesion involving the portacaval lymph node.

**Figure 3:**
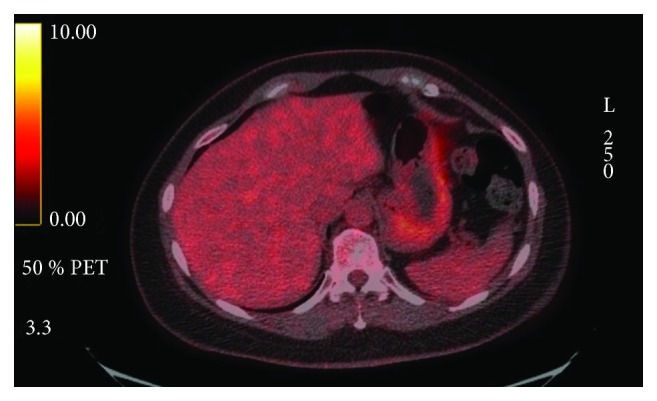
PET/CT after 4 cycles of Pembrolizumab.

The patient was staged as Stage IVa (cT3N2bM0) per AJCC 7th ed. Due to the extension of the parotid disease toward the main trunk of cranial nerve (CN) VII, there was a concern for postoperative CN VII palsy with surgical management. Surgery was therefore deferred, and definitive cisplatin-based concurrent/chemoradiation treatment was initiated. On first surveillance PET/CT, at 12 weeks postconcurrent chemoradiation treatment, she was found to have PET-avid hepatic and bone lesions (Figure 1). A CT-guided portacaval lymph node biopsy confirmed a metastatic disease (Figure 2). IHC staining of the portal cava lymph node demonstrated 100% PD-L1 expression. Next Generation Sequencing was negative for additional mutations. Pembrolizumab monotherapy resulted in a near complete resolution of her hepatic metastasis and complete metabolic resolution of the left parotid mass, cervical adenopathy, and skeletal lesions on PET/CT following four cycles (Figure 3). Follow-up PET/CT scan found a progression of disease per RECIST v1.1 criteria after seven months of treatment.

## 3. Discussion

Salivary gland malignancies are uncommon, accounting for 3-6.5% of all head and neck cancers [3]. Undifferentiated salivary gland malignancies, however, are exceedingly rare. Less than 1% of all salivary gland tumors are lymphoepithelial or undifferentiated carcinoma, carrying a poor prognosis [4]. The reported incidence of undifferentiated carcinoma ranged from 1 to 5.5% in all parotid gland malignancies [5]. A literature search for undifferentiated parotid carcinoma returned seldom results; most of these reports were small case series [6–8]. Furthermore, this patient with poorly differentiated parotid carcinoma behaved more like a nasopharyngeal carcinoma (NPC). Traditionally, salivary gland malignancies are slow growing and become metastatic late in the disease process [1]. Conversely, our patient developed metastatic disease early in her course following cytotoxic chemotherapy and local radiation.

Many features of our patient's case mirror previously reported characteristics of NPC, which may suggest a relationship between NPC, salivary carcinomas, and other EBV-associated malignancies. First, ninety-five percent of primary nasopharyngeal carcinomas are poorly differentiated or undifferentiated, nonkeratinizing carcinomas, with the highest incidence in the Asian and Pacific islands [9]. NPC is also well known to be an EBV-associated malignancy and characteristically causes lymphocytic infiltrates surrounding tumors [9]. Finally, the upregulation of PD-L1 expression has been documented in multiple EBV-associated malignancies. While the exact mechanism of PD-L1 upregulation is not well understood, it is suspected to be due to constitutively activated oncogenic pathways [9–11]. In one study, all patients with high tumor PD-L1 expression (PD-L1 expressed in 90-100% of malignant cells) were positive for EBV, similar to our patient [11]. There is conflicting data on the prognostic value of PD-L1 expression in NPC; however, recent studies seem to suggest that a high percentage of PD-L1 expression may be associated with a poor prognosis [12]. Likewise, in other salivary gland malignancies, higher PD-L1 expression has been associated with a more aggressive cancer [13]. In this patient, the similarities between her histology and disease progression bears a striking resemblance to that seen in NPC, suggesting a similar pathophysiology of malignant transformation in undifferentiated primary parotid carcinoma and aggressive behavior.

Targeted immunologic chemotherapy is emerging as a treatment for metastatic malignancies. Research regarding the use of PD-L1 inhibitors in multiple malignancies such as metastatic renal cell carcinoma, lymphoma, breast, colorectal cancer, nonsmall cell lung cancer (NSCLC), urothelial carcinoma, head and neck squamous cell, and melanoma is currently ongoing [10, 14–17]. These studies continue to elucidate the place of Pembrolizumab in various malignancies. For the treatment of recurrent or metastatic head and neck squamous cell cancers, Pembrolizumab was only recently approved in 2016 by the Food and Drug Administration [2]. Published last year, the KEYNOTE-028 was a multicohort, nonrandomized, phase Ib study of advanced salivary gland tumors with PD-L1 expression undergoing treatment with Pembrolizumab [1]. In this study, 46% of patients experienced stable disease with similar adverse events as reported in prior studies.

Additional trials investigating immunotherapy in recurrent and refractory head and neck cancer are now recruiting, for example, a phase-IIb study by Hsu et al. assessing benefit of using PD-L1 inhibitors for treatment of recurrent or metastatic NPC, which was published after the initiation of this patient's treatment [18]. In the 27 patients treated with Pembrolizumab, there was a promising outcome with an objective response rate of 25% per the RESIST v1.1 [18]; however, they could only confidently say that Pembrolizumab would be safe in this population given low power. It is important to note the growing use of targeted therapy has largely been oncologist driven with patients who have poor prognosis and limited therapeutic options, as seen in this case.

As immunotherapies continue to improve and provide significantly longer progression free and overall survival benefit, genetic sequencing and IHC staining should be incorporated into the work-up of a recurrent or metastatic head and neck malignancies.

Still, additional research is needed to fully understand the role of immunotherapies in rare disease types.
